# Impact of Serum Amyloid A Protein in the Human Breast: An In Vitro Study

**DOI:** 10.3390/nu16142283

**Published:** 2024-07-16

**Authors:** Carolina Dumke de Siqueira, Fátima Regina Mena Barreto Silva, Leandro Borges, Ana Carolina Rabello de Moraes, Elaine Hatanaka, Fabíola Branco Filippin-Monteiro

**Affiliations:** 1 Post-Graduation Program in Pharmacy, Health Sciences Centre, Universidade Federal de Santa Catarina, Florianópolis 88040-900, SC, Brazil; caroldsiqueira@gmail.com; 2 Institute of Cellular Bioelectricity (IBIOCEL): Science & Health, Department of Biochemistry, Biological Sciences Centre, Universidade Federal de Santa Catarina, Florianópolis 88040-900, SC, Brazil; mena.barreto@ufsc.br; 3 Institute of Physical Activity and Sports Sciences, Cruzeiro do Sul University, São Paulo 08060-070, SP, Brazil; sbleandro@yahoo.com.br (L.B.); elaine.hatanaka@unicsul.br (E.H.); 4 Department of Clinical Analysis, Health Sciences Centre, Universidade Federal de Santa Catarina, Florianópolis 88040-900, SC, Brazil; ana.c.moraes@ufsc.br

**Keywords:** breastfeeding, lactation, SAA, human breast cells

## Abstract

The mammary gland is an exocrine gland whose main function is to produce milk. Breast morphogenesis begins in the embryonic period; however, its greatest development takes place during the lactation period. Studies have found the expression of serum amyloid A protein (SAA) in both breast cells and breast milk, yet the function of this protein in these contexts remains unknown. Insufficient milk production is one of the most frequent reasons for early weaning, a problem that can be related to the mother, the newborn, or both. This study aims to investigate the relationship between lactogenesis II (the onset of milk secretion) and the role of SAA in the human breast. To this end, mammary epithelial cell cultures were evaluated for the expression of SAA and the influence of various cytokines. Additionally, we sought to assess the activation pathway through which SAA acts in the breast, its glucose uptake capacity, and the morphological changes induced by SAA treatment. SAA expression was observed in mammary epithelial cells; however, it was not possible to establish its activation pathway, as treatments with inhibitors of the ERK1/2, p38MAPK, and PI3K pathways did not alter its expression. This study demonstrated that SAA can stimulate IL-6 expression, inhibit glucose uptake, and cause morphological changes in the cells, indicative of cellular stress. These mechanisms could potentially contribute to early breastfeeding cessation due to reduced milk production and breast involution.

## 1. Introduction

The mammary gland, originating from the ectoderm, is an exocrine gland whose main function is to produce milk to feed the offspring. Breast morphogenesis begins in the embryonic period; however, its greatest development occurs during the lactation period [1]. The first stage of morphogenesis begins in the uterus, approximately in the seventh gestational week, when the formation of the rudimentary duct begins, and it develops until puberty, the phase in which terminal buds will be formed by the release of estrogen. During adulthood, the ducts become more branchy, and it is at the beginning of pregnancy that the formation of lobuloalveolar structures begins in response to progesterone stimulation. During pregnancy, these structures mature, and the lactation phase is the period in which they are completely developed [2].

While in the breast of non-pregnant adult women, connective and adipose tissue is predominant and epithelial tissue is scarce, the beginning of pregnancy is marked by both organizational and functional changes in the mammary gland, in which there is a proliferation of ductal and alveolar cells, promoting breast growth, concomitant with the reduction of adipose tissue [3]. This development leads to the formation of an extensive branched ductal system and a multitude of alveoli derived from terminal buds [4].

Serum Amyloid A Proteins (SAAs) are made of up to 104 amino acids and are related to the acute phase response. It can increase their concentration up to a thousand folds in 24 h, especially in situations such as inflammation, trauma, infection and even cancer [5]. In humans, these proteins can be classified into SAA1, SAA2, SAA3 and SAA4 groups, with SAA1 and SAA2 further subdivided into SAA1α, SAA1β, SAA1γ, SAA2α and SAA2β; their structures and functions differ from each other. Several functions have already been described for SAAs, but the general function is still unclear. It was found to be related to the migration of leukocytes through the induction of chemokines, the chemoattraction of monocytes [6] and immature dendritic cells, neutrophils and T cells [7]. Furthermore, it also has the potential to induce cytokines and M2-type macrophages, and has a role in cholesterol transport, among other things [8].

In the mammary tissue, only the expression of SAA1, SAA2 and SAA4 and not SAA3 was seen; however, Larson et al. detected the expression of the human SAA3 gene using RT-PCR after the stimulation of mammary gland epithelial cells with lactation hormone prolactin (PRL) or the acute phase stimulating LPS [9].

It was previously suggested that the effect of SAAs on the proliferation of a preadipocyte cell line (3T3-L1) was mediated by the ERK1/2 signalling pathway; however, it was not possible to elucidate the roles of the PI3K and p38MAPK pathways. Another important finding was the inhibition of the expression of GLUT4 and the stimulation of the pro-inflammatory cytokines (IL-6 and TNF-α) through this protein [10]. Altogether, this study aimed to analyze the function of serum amyloid A protein in the ERK1/2, p38MAPK and PI3K pathways and in the glucose receptor pathway in the human breast through cellular models to elucidate possible factors associated with an early decrease in lactogenesis and the terms effect of breastfeeding.

## 2. Materials and Methods

### 2.1. Cell Culture

The human breast cell line MCF-10A (ATCC CRL-10317) (Manassas, VA, USA) was grown in plastic cell culture bottles containing Dulbecco’s Modified Eagle’s Medium (DMEM)/Ham’s Nutrient Mixture F12 (1:1) (Sigma-Aldrich, Milwaukee, WI, USA), 100 U/mL penicillin, 100 μg/mL streptomycin and 10 mM HEPES, and supplemented with 2.5 mM L-glutamine, 5% horse serum, 10 µg/mL human insulin, 0.5 µg/mL hydrocortisone and 10 ng/mL EGF. The cells were maintained in a humidified incubator at 37 °C, in an atmosphere of 5% carbon dioxide (CO_2_).

### 2.2. Cell Viability

The 3-(4,5-dimethyl-2-thiazolyl)-2,5-diphenyl-2H-tetrazolium bromide (MTT) assay was used to evaluate cell viability in response to specific pharmacological inhibitors of ERK1/2 (PD98059), p38MAPK (SB203580) and PI3K (wortmannin). To evaluate the cytotoxicity of these inhibitors to the human breast cell line MCF-10A, cells were plated at a density of 5 × 10^3^ cells per well in 96-well plates. After 48 h of plating, the culture medium was changed and, after 96 h, the cells were maintained in culture medium with 0.5, 5 and 10% horse serum. After 144 h of plating, the cells were treated with the inhibitors.

Cells incubated with the culture medium alone were used as a control. After a period of 48 h of treatment, the culture medium in the wells was replaced with an MTT solution with the culture medium for 2 h, and it was then removed and 100 μL of dimethyl sulfoxide was added to dissolve the crystals. To determine the concentration of MTT reduced to formazan by the mitochondrial dehydrogenases of the viable cells, the absorbance was measured on a Perkin-Elmer LS55 spectrophotometer (Boston, MA, USA) at a wavelength of 540 nm. This experiment was carried out in triplicate on three consecutive days.

### 2.3. Cell Proliferation Assay

For the cell proliferation assay, the same specific pharmacological inhibitors of ERK1/2 (PD98059), p38MAPK (SB203580) and PI3K (wortmannin) were used. For this, MCF-10A cells were plated at a density of 3 × 10^4^ cells per well in 24-well plates. After 48 h of plating, the culture medium was changed and, after 96 h, the cells were maintained in serum-deprived culture medium (0.5% horse serum). After 144 h of plating, the cells were treated with the inhibitors alone at concentrations of 50 μM (PD98059), 10 μM (SB203580) and 100 nM (wortmannin) or in association with 5 μg/mL of rSAA. After 48 h of treatment, the cells were trypsinized and cell proliferation was assessed by counting cells using the Neubauer chamber. This experiment was carried out in triplicate on three consecutive days.

### 2.4. Clonogenic Assay

Cells previously treated with 5 μg/mL rSAA, 50 μM PD98059, 10 μM SB203580 and/or 100 nM wortmannin were seeded in 6-well plates at a density of 500 cells/well. The cells were maintained in culture for another seven days at 37 °C in an atmosphere containing 5% CO_2_, and the culture medium was changed every 48 h. After the 7th day, when the cells had formed sufficiently large colonies, the medium was removed from the wells and the cells were washed carefully with 1× phosphate-buffered saline (PBS). Subsequently, the PBS was removed, and the cells were fixed with 6% glutaraldehyde. After fixing the cells, a 0.5% crystal violet solution was added to each well for 30 min to stain the colonies. After incubation, the wells were washed with water and air-dried at room temperature (21 °C). After the wells were completely dry, digital images of the colonies were obtained using a camera. The area of the colonies was analyzed using the ImageJ software (V1.51p) and the ColonyArea plugin [11]. This experiment was carried out in triplicate on three consecutive days.

### 2.5. Enzyme-Linked Immunosorbent Assay in Cell Supernatant

Cell supernatant samples were collected and stored at −80 °C before the determination of markers by enzyme-linked immunosorbent assay (ELISA). The concentrations of IL-6, MCP-1, IFN-γ and SAA were determined by ELISA, according to the instructions of the manufacturer BD OptEIA™ (Franklin Lakes, NJ, USA) for the analyses of IL-6, MCP- 1 and IFN-γ, and Invitrogen™ (Camarillo, CA, USA) for SAA analysis. The assays were carried out according to the manufacturer’s instructions.

### 2.6. ^14^C-Glucose Uptake

MCF-10A cells were plated at a density of 5 × 10^3^ cells per well in 96-well plates. After 48 h of plating, the culture medium was changed and, after 96 h, the cells were maintained in 0.5% horse serum culture medium. After 144 h of plating, the cells were treated with 5 µg/mL of rSAA and an untreated control was also maintained. After 48 h of treatment, the culture medium was removed, and all wells were washed with 1× PBS. Then, 100 µL of Krebs Ringer-bicarbonate (KRb) (122 mM NaCl, 3 mM KCl, 1.2 mM MgSO_4_, 1.3 mM CaCl_2_, 0.4 mM KH_2_PO_4_, 25 mM NaHCO_3_) was added to the wells, with ^14^C-DG (0.1 μCi/mL), and incubated for 30 min at 37 °C. After incubation, all liquid was removed, and all wells were washed again with 1× PBS to remove the excess radioactive material. Then, 200 µL of 0.5 M NaOH was added for cell lysis and of this quantity, 25 µL was used and dissolved in 1 mL of scintillation liquid [12]. The remaining samples were used for protein determination using the Lowry method. The samples were kept in the refrigerator until the reading was performed using a Tri-Carb^®^ 2800TR LSA scintillator (Perkin Elmer, Waltham, MA, USA). The protein dosage of the samples used for the glucose uptake assay was measured according to Lowry et al., 1951 [13]. The results were expressed as nmol of glucose/mg of protein.

### 2.7. Transmission Electron Microscopy (TEM)

MCF-10A cells were plated at a density of 3 × 10^4^ cells per well in 24-well plates. After 48 h of plating, the culture medium was changed and, after 96 h, the cells were maintained in serum-deprived culture medium (0.5% horse serum). After 144 h of plating, cells were treated with 5 μg/mL rSAA and an untreated control was also maintained.

After 48 h of incubation with the treatments, the cells were trypsinized, transferred to microtubes and centrifuged to remove all the culture medium and treatments. Afterwards, they were fixed using a fixative solution of 4% paraformaldehyde, 3% glutaraldehyde and 0.1 M sodium cacodylate. After 24 h in a fixative solution, cells were washed 3 times with 0.1 M sodium cacodylate buffer and then fixed with 1% osmium tetroxide for 2 h. The cells were then washed again 3 times with 0.1 M sodium cacodylate buffer and subsequently dehydrated with increasing concentrations of alcohol. After the dehydration stage, the infiltration process began, with increasing concentrations of resin. After the last infiltration step, the resin containing the cell pellet was polymerized at 70 °C for 48 h. Afterwards, ultra-thin sections containing the cells were made and placed on the grid, contrasted with 5% uranyl acetate and lead citrate to be read using a 100 kV transmission electron microscope (JEM 1011, JEOL, Peabody, MA, USA).

### 2.8. Data Analysis

Numerical results were expressed in mean and standard deviation and the symmetry of the data was assessed using the Shapiro–Wilk test [14]. Differences between groups were evaluated using the one-way ANOVA test with the Bonferroni post-test when three or more groups were compared, and Student’s *t*-test was used when comparing two groups. The level of significance was set at *p* ≤ 0.05 for all analyses. All analyses were performed with GraphPad Prism5 (GraphPad Software, San Diego, CA, USA).

## 3. Results

Considering that adipose tissue and the mammary gland are adjacent tissues, it was proposed that SAA signalling in breast cells occurs through the ERK1/2, p38 MAPK and PI3K/Akt pathways, in the same way as in adipocytes.

To evaluate the cytotoxicity of the inhibitors and the cell viability in different concentrations of horse serum, an MTT assay was performed. After analyzing cell viability, it was seen that all cells showed viability above 80%, regardless of the treatment or serum concentration; therefore, inhibitors could be used at concentrations of 50 μM (PD98059), 10 μM (SB203580) and 100 nM (wortmannin), and a horse serum concentration of 0.5% was also chosen for subsequent experiments.

**Figure 1:**
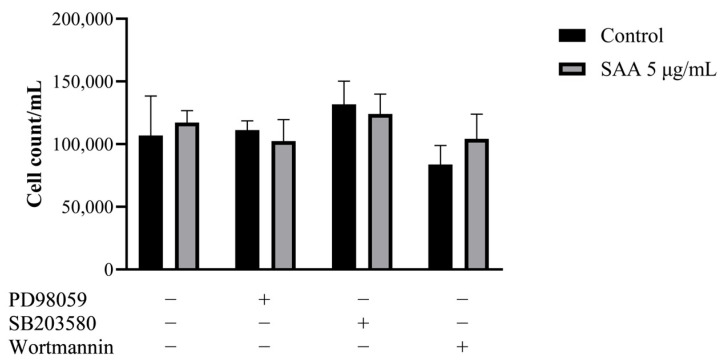
Results were expressed as mean and standard deviation after 48 h of treatment incubation. PD98059 50 μM, SB203580 10 μM and wortmannin 100 nM or in association with 5 μg/mL of rSAA. SAA, serum amyloid A protein.

The cell proliferation assay was carried out to evaluate whether inhibitors, in the presence of SAA, could reduce mammary cell proliferation, thus indicating that SAA would possibly act on the pathway that was being inhibited. However, it was seen that there was no significant decrease in proliferation in any of the tested pathways (Figure 1), suggesting that SAA does not act directly on any of the three pathways (ERK1/2, p38 MAPK and PI3K).

**Figure 2:**
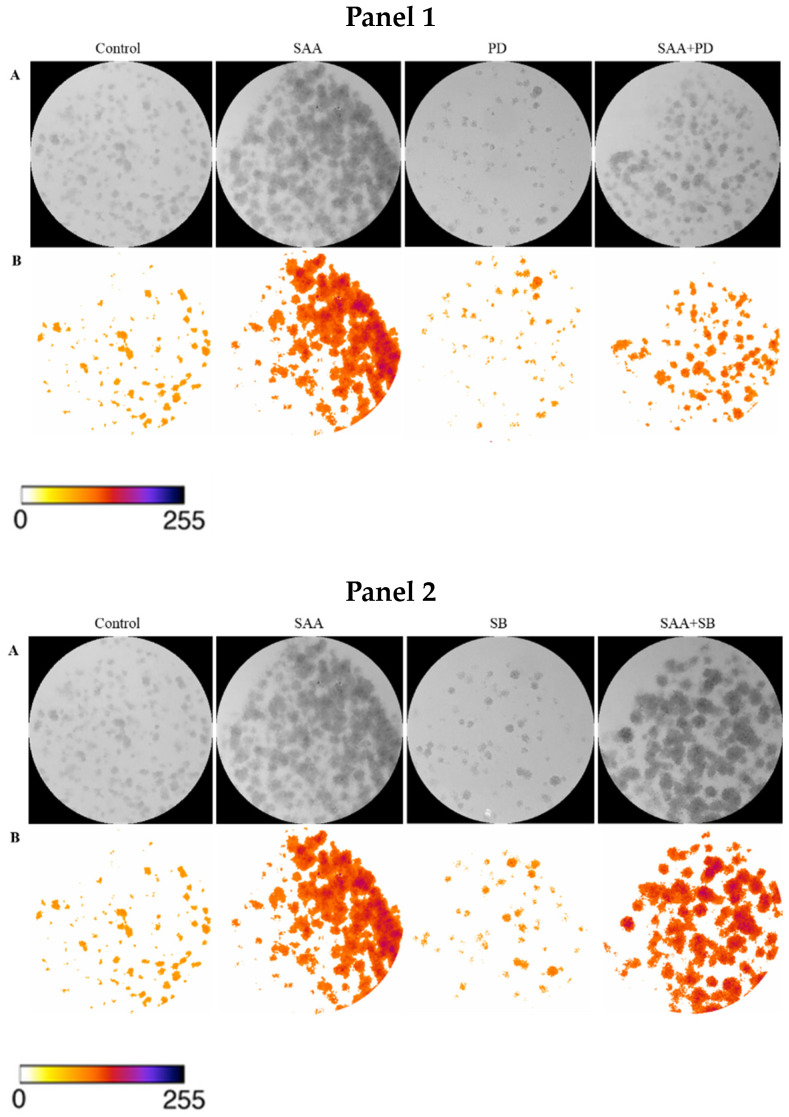

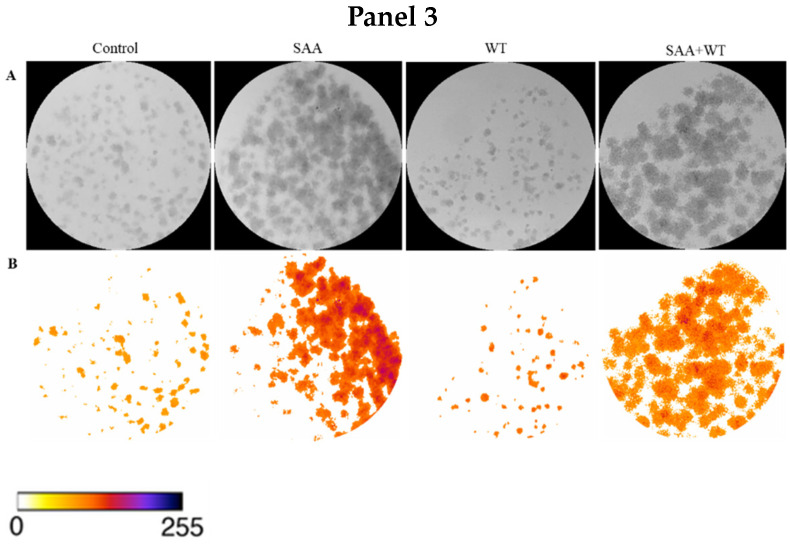
Images obtained using ImageJ software: (**A**) 8-bit grayscale images of individual wells demonstrating different levels of colony formation according to each treatment. (**B**) Same individual wells after thresholding and background removal by the “Colony_thresholder” macro. Panel 1: Control—no treatment; SAA—treatment with SAA protein 5 μg/mL; PD—treatment with the inhibitor PD98059 50 μM; SAA + PD—treatment with SAA 5 μg/mL and the inhibitor PD98059 50 μM; Panel 2: Control—no treatment; SAA—treatment with SAA protein 5 μg/mL; SB—treatment with the inhibitor SB203580 10 μM and SAA + SB—treatment with SAA 5 μg/mL and the inhibitor SB203580 10 μM; Painel 3: Control—no treatment; SAA—treatment with SAA protein 5 μg/mL; WT—treatment with wortmannin inhibitor 100 nM; SAA + WT—treatment with SAA 5 μg/mL and the inhibitor wortmannin 100 nM. The colour bar represents the intensity scale displayed in the thresholded wells. Zero intensity (white) corresponds to areas where no cells were identified (background).

**Figure 3:**
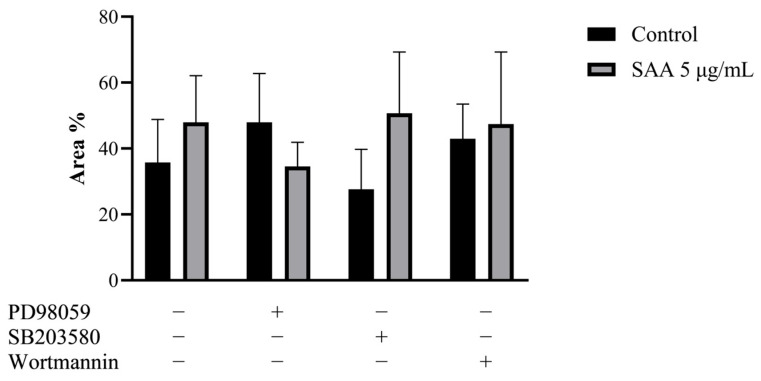
Results expressed as a percentage of occupied area and standard deviation. Control samples refer to samples without treatment and SAA refers to samples treated with SAA 5 μg/mL. Cells were also treated with specific pharmacological inhibitors of ERK1/2 (PD98059 50 μM), p38MAPK (SB203580 10 μM) and PI3K (wortmannin 100 nM). One-way ANOVA with Bonferroni post-test.

To confirm the result obtained in the cell proliferation assay, the clonogenic assay was carried out, in which the ability of cells treated or not with inhibitors to survive and grow, producing colonies, was evaluated. The images taken from each well were analyzed in ImageJ software concerning colour intensity (Figure 2) and the percentage of the well area occupied by the colony (Figure 3).

Corroborating the results found from the cell proliferation assay, there was no significant difference between the different treatments, demonstrating that there was no difference in the cell’s ability to produce colonies even when treated with SAA and inhibitors.

**Figure 4:**
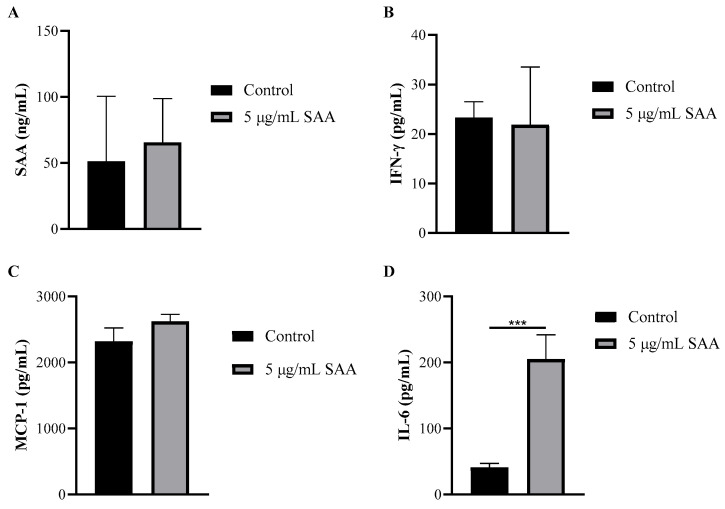
Concentration of (**A**) SAA, (**B**) IFN-γ, (**C**) MCP-1 and (**D**) IL-6 in cell supernatant after 48 h of treatment incubation. Results were expressed as mean and standard deviation. SAA, serum amyloid protein A; IFN-γ, interferon-gamma; MCP-1, monocyte chemoattractant protein 1; *** *p* ≤ 0.0005 compared to the respective control group.

**Figure 5:**
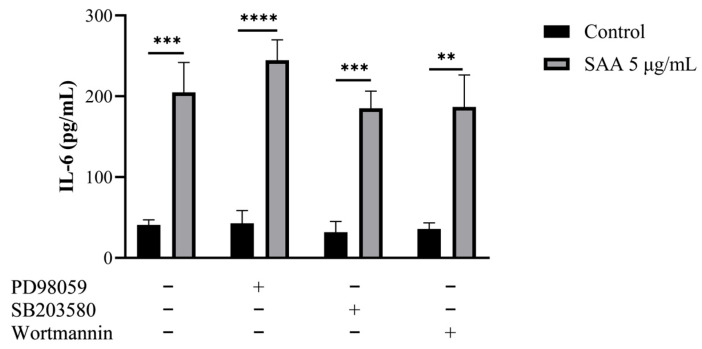
Results were expressed as mean and standard deviation after 48 h of treatment incubation. Control samples refer to samples without treatment and SAA (serum amyloid protein A) refers to samples treated with SAA 5 μg/mL. Cells were also treated with specific pharmacological inhibitors of ERK1/2 (PD98059 50 μM), p38MAPK (SB203580 10 μM) and PI3K (wortmannin 100 nM). One-way ANOVA with Bonferroni post-test; ** *p* ≤ 0.001; *** *p* ≤ 0.0005; **** *p* ≤ 0.0001 as compared to respective control groups.

Using the cell culture supernatant, the concentrations of inflammatory markers were evaluated after SAA stimulation using the ELISA method (Figure 4). The concentration of IL-6 was also evaluated when stimulated by SAA and by the inhibitors PD98059, SB203580 and wortmannin (Figure 5).

Through this assay, it was seen that breast cells express SAA even when not stimulated. Another important finding was that all cells treated with SAA showed higher concentrations of IL-6 in the supernatant compared to untreated cells.

**Figure 6:**
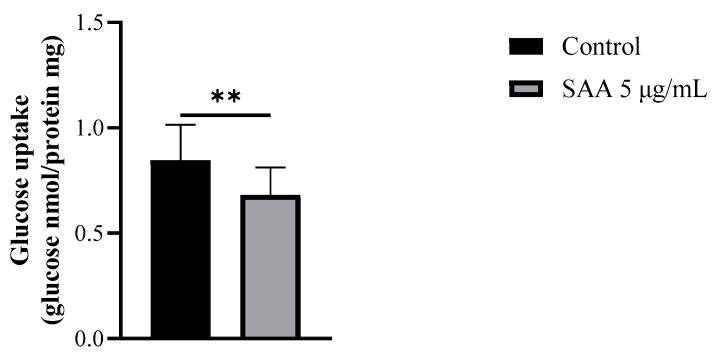
Results expressed as mean and standard deviation after 48 h of treatment incubation. Control samples refer to samples without treatment and SAA (serum amyloid protein A) refers to samples treated with SAA 5 μg/mL. Mann–Whitney-U test; ** *p* = 0.0071.

Considering that the inhibitors used did not interact with SAA in the previous results, it was decided to continue the experiments without them. Therefore, to evaluate the cellular glucose uptake capacity after treatment with SAA, the glucose uptake assay was performed on breast cells treated with 5 μg/mL SAA (Figure 6). As a result, cells treated with SAA showed a decrease in glucose uptake capacity.

**Figure 7:**
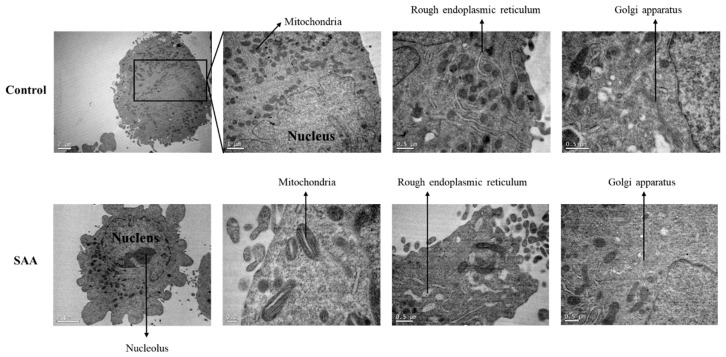
Representative photomicrographs of ultrathin sections after 48 h of treatment incubation. Electron microscopy of MCF-10A cells. Control samples refer to samples without treatment; SAA (serum amyloid protein A) refers to samples treated with SAA 5 μg/mL.

To evaluate the morphological changes in the cells following treatment with SAA, a transmission electron microscopy assay was performed (Figure 7). Through analysis of the cells by transmission electron microscopy, a wide distribution of mitochondria and rough endoplasmic reticulum with regular morphologies was noticed in control cells without treatment. However, when cells were treated with SAA, mitochondria had an altered morphology, displaying a central lumen. The rough endoplasmic reticulum also had an increased lumen with respect to control cells.

## 4. Discussion

In this study, it was possible to detect the presence of SAA in mammary epithelial cells; however, there is still a gap in the literature regarding its function and mechanism of action. As far as we know, this is the first study that attempts to demonstrate the role of SAA in the human breast. Since the mammary gland has a close association with the deposition of mammary adipose tissue, and since adipocytes are important local regulators of the growth of mammary epithelial cells and also their function during lactation [15], it has been proposed that SAA acts in the human breast in the same signalling pathways that act in adipose tissue (ERK1/2, p38 MAPK and PI3K/Akt). Another relevant piece of information for this theory is that cytokines regulate cell proliferation and inflammation in the breast through the MAPK and PI3K pathways [16]. Therefore, in this study, the proliferation capacity of mammary epithelial cells was tested after the inhibition of three possible SAA signalling pathways in the breast, the ERK1/2, p38 MAPK and PI3K/Akt pathways, as well as the SAA expression in response to the inhibition of its pathways.

Firstly, the inhibitors were tested for their cytotoxicity, for which a cell viability assay was carried out. Along with this assessment, whether serum deprivation would lead to cell death was investigated. According to the study by Kues and collaborators, serum deprivation has effects on the cell cycle, allowing cycle synchronization, so that most cells are in the G0/G1 stage [17]. At the end of this experiment, cell viability greater than 80% was observed regardless of the treatment used; therefore, the cell proliferation experiment was carried out using pre-established concentrations of inhibitors and SAA, as well as the lowest tested serum concentration (0.5%). Both in the cell proliferation assay, through counting viable cells, and in the clonogenic assay, in which it was possible to evaluate the capacity of treated cells to form colonies, no significant differences were found between the groups; therefore, SAA, possibly, does not act on pro-proliferation signalling of any of the three pathways investigated.

However, when evaluating the expression of cytokines in the cell supernatant in the different treatments, it was seen that when treating the cells with SAA there was a greater expression of IL-6. In breast tissue, Zhao, Melenhorst and Hennighausen demonstrated that IL-6 contributed to the induction of controlled remodelling during the involution phase, in part through the MAPK pathway. They also demonstrated that IL-6 expression is induced in breast tissue at early stages of involution, suggesting that it is an inducer of breast remodelling and that this involution is mediated by Stat3 [18]. The transcription factor Stat3 is activated in mammary tissue after weaning; consequently, an inactivation of Stat3 in the alveolar epithelium results in a delay in tissue remodelling during involution [19]. Therefore, SAA would not necessarily be related to the development and proliferation of breast cells, but perhaps to their remodelling, through activation by IL-6.

In addition to analyzing the concentration of IL-6 in the cell supernatant, we sought to evaluate the capacity of SAA to uptake glucose in mammary cells. As a result, SAA was able to inhibit glucose uptake in MCF-10A cells. It is known that, upon entering the mammary cell, glucose is conjugated with UDP-galactose through lactose synthase, resulting in the production of lactose, which, in addition to being one of the main components of breast milk, also determines the volume of milk according to its concentration; therefore, the entry of glucose into the cell and, consequently, the production of lactose, is responsible for the amount of milk produced by the nursing mother [20]. The studies by Oliveira and collaborators and Monteiro and collaborators demonstrated the effect of inhibiting glucose uptake induced by SAA in adipocytes through the reduction in GLUT4, consequently decreasing glucose transport [10,21]. In the present study, it was not possible to evaluate the expression of GLUTs to identify by which mechanism this inhibition occurs; however, it is believed that it is possibly due to the decrease in GLUT1 and/or GLUT8, since both carry out glucose transport in the breast [22].

Finally, possible morphological changes caused in breast cells after treatment with SAA were evaluated using an electron microscopy test. The images obtained through the assay for control samples were similar to those described by Underwood and collaborators [23]. The cells had abundant mitochondria and rough endoplasmic reticulum, the latter necessary for milk production during lactation. Also, invagination of the nucleus and smooth endoplasmic reticulum and Golgi apparatus were present [23].

When treated with SAA, cells showed some signs of cellular stress, such as the discontinuation of mitochondrial cristae and increased lumens in the rough endoplasmic reticulum. Mitochondria from rat cells treated with ketamine, which has neurotoxic potential, showed similar structures when compared to the cells of this study treated with SAA [24]. In another study, carried out by Kosta and collaborators, the structure of mitochondrial cristae was also remodelled after nutrient deprivation in cells of Dictyostelium discoideum strains, even without cell death [25]. Regarding the endoplasmic reticulum, some studies reported the same variation in morphology in different situations and cell types, such as lipotoxic cell death in ovarian cells [26], hypoxia in mouse brains [27] and ischemia in rat brains [28].

## 5. Conclusions

In conclusion, this study showed that SAA can stimulate the expression of IL-6, inhibit glucose uptake, and cause morphological changes in the cells, possibly indicating cellular stress and a decrease in energy production. Therefore, it could be related to the early decrease in breastfeeding due to reduced breast milk production, as well as breast involution.

## Data Availability

The data used to support the observations of this study are available from the corresponding author upon request.
